# Nutrients Composition in Fit Snacks Made from Ostrich, Beef and Chicken Dried Meat

**DOI:** 10.3390/molecules23061267

**Published:** 2018-05-25

**Authors:** Żaneta Zdanowska-Sąsiadek, Joanna Marchewka, Jarosław Olav Horbańczuk, Agnieszka Wierzbicka, Paulina Lipińska, Artur Jóźwik, Atanas G. Atanasov, Łukasz Huminiecki, Aleksander Sieroń, Karolina Sieroń, Nina Strzałkowska, Adrian Stelmasiak, Stefaan De Smet, Thomas Van Hecke, Louwrens C. Hoffman

**Affiliations:** 1 Institute of Genetics and Animal Breeding, Polish Academy of Sciences, Jastrzebiec, 05-552 Magdalenka, Poland; z.sasiadek@ighz.pl (Ż.Z.-S.); lipinskap.igab.pas@gmail.com (P.L.); aa.jozwik@ighz.pl (A.J.); a.atanasov.mailbox@gmail.com (A.G.A.); l.huminiecki@ighz.pl (Ł.H.); n.strzalkowska@ighz.pl (N.S.); 2 Department of Technic and Food Development, Faculty of Humane Nutrition and Consumer Sciences, Warsaw University of Live Science, 02-787 Warszawa, Poland; agnieszka_wierzbicka@sggw.pl (A.W.); adrian_stelmasiak@sggw.pl (A.S.); 3 Department of Pharmacognosy, University of Vienna, 1090 Vienna, Austria; 4 Department of Internal Diseases, Angiology and Physical Medicine, Center for Laser Diagnostics and Therapy, Medical University of Silesia, Batorego Street 15, 41902 Bytom, Poland; sieron1@o2.pl; 5 Department of Physical Medicine, School of Health Sciences in Katowice, Medical University of Silesia in Katowice, ul. Poniatowskiego 15, 40-055 Katowice, Poland; ksieron@hot.pl; 6 Laboratory of Animal Nutrition and Animal Product Quality, Department of Animal Sciences and Aquatic Ecology, Ghent University, Coupure Links 653, B-9000 Gent, Belgium; Stefaan.DeSmet@ugent.be (S.D.S.); Thomas.VanHecke@UGent.be (T.V.H.); 7 Department of Animal Sciences, Faculty of AgriSciences, University of Stellenbosch, Matieland 7602, South Africa; lch@sun.ac.za

**Keywords:** nutrients, dried meat, heme iron, nutrients, fit snack

## Abstract

The aim of the study was to compare three types of meat snacks made from ostrich, beef, and chicken meat in relation to their nutrients content including fat, fatty acids, heme iron, and peptides, like anserine and carnosine, from which human health may potentially benefit. Dry meat samples were produced, from one type of muscle, obtained from ostrich (*m.* *ambiens*), beef (*m. semimembranosus*), and broiler chicken meat (*m. pectoralis major*). The composition of dried ostrich, beef, and chicken meat, with and without spices was compared. We show that meat snacks made from ostrich, beef, and chicken meat were characterized by high concentration of nutrients including proteins, minerals (heme iron especially in ostrich, than in beef), biologically active peptides (carnosine—in beef, anserine—in ostrich then in chicken meat). The, beneficial to human health, *n*-3 fatty acids levels differed significantly between species. Moreover, ostrich jerky contained four times less fat as compared to beef and half of that in chicken. In conclusion we can say that dried ostrich, beef, and chicken meat could be a good source of nutritional components.

## 1. Introduction

In the last decade, consumer’s interest in snack food products made of dried meats has been growing. This trend has been encouraged by the recommendations made by dietitians to ingest increased amounts of proteins while reducing levels of carbohydrates in meals [1]. Due to the acceleration of lifestyle, particularly observed in Western societies, the demand for easy to prepare and ready to eat food has increased, resulting in a wider selection of these meals and meat snacks on the market [2]. Meat snack sticks have been developed as premium quality products, characterized by a high concentration of nutrients, as well as having high value as a handy and “on the go” food product, dedicated especially to young, physically- and mentally-active people, as well as professional athletes. The main types of meat used as a meat snack sticks constitute beef, especially in USA, and also chicken and game meat [2]. The beef meat is characterized by the high quality of the protein, iron, and vitamin B [3,4,5], whereas chicken meat is relatively low in fat, rich in high quality protein and polyunsaturated fatty acids [6]. Interestingly, among game meat, ostrich is gaining in popularity since it has been recognized as a dietetic, tasty product [7,8,9,10,11,12,13]. In South Africa, snacks produced from this dried meat are called biltong [14,15]. Recently, Poland, who is a leader in the ostrich industry in Europe [16,17,18,19], started to produce snacks from ostrich meat [20]. However, the knowledge regarding the nutritive value of such snacks, especially meat made from ostrich meat is still limited. We hypothesise that dried ostrich, beef, and chicken meat could be good sources of nutrients, especially of heme iron, omega-3 fatty acids, and some other nutritional constituents, with potential benefits for human health. Thus, the aim of current study is to compare three types of meat snacks made from ostrich, beef, and chicken meat in relation to their nutrients including anserine, carnosine, heme iron content, and fatty acid (FA) profile.

## 2. Materials and Methods

### 2.1. Preparation of the Dry Meat Samples

Dry meat samples were produced according to the sampling protocol described by reference [20], from one type of muscle, obtained from nine individuals per each species: Ostrich (*m. ambiens*), beef (*m. semimembranosus*), and broiler chicken meat (*m. pectoralis major*). Meat, trimmed of all visible connective tissue, was submerged in brine for 48 h. The brine was composed of 2% NaCl, 0.5% NaNO_2_, 0.5% cayenne pepper extract, and 97% of water.

Samples within the species were assigned into three treatment groups: Control (NO: natural ostrich, NB: natural beef and NCh: natural chicken) to which no additives were added; salted (SO: salted ostrich, SB: salted beef, SCh: salted chicken) with 5% of sea salt in flakes; and spices (SpO: spicy ostrich, SpB: spicy beef, SpCh: spicy chicken) with 14% of dried tomatoes and 1% of each pepper: Black, red, green, and white. Spices were added after dripping off excess brine. Thereafter, each meat sample was divided equally into three parts, each part cut into 6–7 mm thick slices, perpendicular to the muscle fibers, providing nine samples in each treatment group. The spiced treatment meat slices were surrounded by dry spices. Each meat part was placed on dryer shelves and dried for 17 h at 50 °C under forced air (average flow velocity 2.5 ± 0.5 m/s). The meat was then cooled for 2 h to 20 °C in dry air. Dried meat slices were packed into 50 g bags, with a separate bag for each of the three samples from each species and treatment and stored in an anaerobic atmosphere. The whole described procedure was repeated in three replicates using different individuals in each of them. Authors of current work developed and patented previously the preparation method of a homogeneous dried meat jerky commercial product [16]. Based on that experience it was known that between samples variability of the key dried meat characteristics was low (coefficient of variation below 5%), which justifies the sample size of three individuals and three replications, giving in total sample size *n* = 9 in each treatment group in current study.

### 2.2. Chemical Composition of the Meat Samples

Prior to analysis, the pieces of dried meat were thoroughly ground.

Meat samples were analyzed for dry matter, crude protein, and crude fat contents according to the relevant ISO 1442-1973, ISO 937-1978, and ISO 1444-1973 methods, respectively. The meat sample of 200 g was homogenised using mechanical homogeniser T18 Ultra-Turrax (IKA^®^ Works Inc., Cincinnati, OH, USA) which included a high speed rotation cutter. The crude protein analysis was performed according to ISO 1442/1973 method of Kjeldahl (estimation of the total nitrogen), using K-375 KjelMasters System and Scruber K-415 Butchi Laborteknik AG Switzerland. The crude fat was analysed using the Soxhlet’s system (extraction with petroleum ether) using E461 Butchi Laborteknik AG Switzerland. Dry matter was analysed using the laboratory drier at the 103 °C ± 2 °C (Memert, GmbH & Co., Düren, Germany). Using the oven Nabertherm, series L (Nabertherm GmbH, Lilienthal, Germany), the crude ash was estimated with the method ISO 936:2000 at the temperature 550 °C ± 25 °C.

Hematin was determined colorimetrically using the method of Hornsey (1956) [21] and converted to heme-Fe using the formula:

heme-Fe = hematin × atomic weight Fe/molecular weight hematin.

Carnosine and anserine concentration were determined by high performance liquid chromatography (HPLC) using the method of Kobe, Ishihara, Takano, & Kitami (2011) [22]. One gram of ground dry meat was weighed and extracted in a 0.01 M phosphate buffer at pH 7.4. After homogenization, 2.0 mL of mixture was collected. To each sample, 1.0 mL of acetonitrile was added, mixed, kept overnight at 4 °C, and centrifuged at 3000 rpm for 10 min. The supernatant was then filtered over a cellulose syringe filter of 0.20 µm and transferred to a vial. The HPLC system used was an Agilent Technologies 1200 Series (2006, Santa Clara, CA, USA) coupled to a diode-array detector (DAD). Twenty µl of sample was injected and dipeptides were determined by isocratic separation at a flow rate of 1.2 mL/min at 30 °C for 26 min. Detection was by means of UV absorption at 210 nm. Carnosine was eluted at a RT (rotation time) of ~16 min, followed by anserine (RT ~ 21 min). Concentration determination was carried out by comparing with standard solutions of both anserine and carnosine with known concentrations between 0.02 and 0.10 mg/mL.

For fatty acid analysis, lipids were extracted in duplicate from 2 g meat samples by means of a modification of the chloroform/methanol (2/1; *v*/*v*) method of Folch, Lees, & Sloane-Stanley (1957) [23]. After methylation of the fatty acids with NaOH/MeOH followed by HCl/MeOH, the fatty acid methyl esters (FAME) were analysed by gas-liquid chromatography (HP 6890) using a CP Sil88 column for FAME (100 m × 250 mm × 0.25 µm) (Chrompack, Middelburg, The Netherlands). The GC conditions were: injector: 250 °C; detector: 280 °C; H_2_ as carrier gas; temperature program: 150 °C for 2 min, followed by an increase of 1.5 °C/min to 200 °C, then 5 °C/min to 215 °C. Peaks were identified by comparing the retention times with those of the corresponding standards (Sigma, Overijse, Belgium: Nu-Chek Prep., Waterville, MN, USA).

### 2.3. Statistical Analysis

A generalised linear mixed model analysis was performed on all measured parameters, including “species”, “additive”, and their interaction as fixed factors. The validity of the models was tested by using Akaike’s information criterion. PROC GLIMMIX of SAS v 9.3 (SAS Institute Inc., Cary, NC, USA) including the Tukey adjustment option was used to conduct the analysis. The least square means for all significant effects in the models (*p* ≤ 0.05) were computed using the LSMEANS option. The trend of a significant effect was considered for *p* < 0.10.

## 3. Results and Discussion

### 3.1. Chemical Composition

The chemical composition and heme iron content of the dried ostrich, beef, and chicken meat are presented in the Table 1. There was no effect of the interaction between species and additives applied on the dry matter content. Dry matter content was highest in ostrich jerky meat compared to beef and chicken (Table 1). Despite the differences in dry matter content between the three species in the current study, the jerky meat was characterized by the optimal water content for dried meat products (below 15%) [15,24], protecting the meat from quality deterioration over storage time [25]. Dry matter was highest in the salted jerkies compared to those spiced, an expected result since salt is known to dehydrate the meat tissue in dried products [26]. The protein content in jerky meat was affected by a species additive interaction (Table 1), where the highest levels were observed in natural and salty ostrich, as well as salty chicken jerky meat, whereas the lowest was measured in natural and spicy beef. Natural and salty ostrich jerky meat was also characterized by the highest dry matter content. Comparable protein contents ranging from 20 to 22% has been reported in raw ostrich meat [10], raw beef [27], and raw chicken [6,28].

Overall, the lowest fat content was observed in ostrich jerky—four times lower than compared to beef and half of that in chicken (Table 1). The fat content in jerkies differed in beef and chicken depending on the applied additive, while additive had no effect in ostrich meat. Previously, ostrich raw meat was reported to have lower fat (1.2%) content than beef (4.5%) [29] and chicken meat (3.0%) [30]. The lower fat content of dried ostrich meat compared to beef and chicken jerkies indicates it to be of a high nutritive- and low caloric value.

Meat is a valuable source of heme iron in the human diet [31,32]. Overall, the ostrich meat jerky (8.6 mg/kg) was twice as rich in heme iron as beef and twenty times as chicken jerky (Table 1). The concentrations of heme iron in the current study were dependent on species by additive interaction (Table 1). Addition of salt followed by spices significantly decreased the heme iron content in ostrich dried meat down to 18%, since the weight of the spices was included in the total weight of the product. Previously, ostrich, beef, and horse raw meat were reported to contain high concentrations of iron and heme iron [19,33], while after applying high temperature, levels of iron and heme iron remained on a similar level in the ostrich meat, while decreasing by up to twenty percent in beef, lamb, and pork [34]. High temperatures also reduced the iron bioavailability [35]. Therefore, it is crucial to apply appropriate temperatures during meat processing [33]; it should not be higher than 55 °C [36], to avoid myoglobin denaturation. In the current study, an applied temperature of 50 °C provided optimal stability for the technology of the production of the dried snacks made of the three types of the meat: Ostrich, beef and chicken (P. P.414678) [20]. Furthermore, the applied drying procedure provided slow evaporation of the moisture from the meat slices, as well as equalization of humidity levels of meat slices with dried spices and proper bonding of the dried slices with the spices.

### 3.2. Carnosine and Anserine Content

The concentrations of the biologically active peptides—carnosine and anserine differed between beef, chicken, and ostrich dry meat samples, but not according to applied additives (Table 1). Ostrich meat proved to be very rich in anserine (16.8 ± 0.701 mg/g) as compared to the other meats, especially beef (2 ± 0.103 mg/g), on the other hand, beef had the highest carnosine content (Table 1). The addition of the spices significantly decreased both the content of carnosine, as well as anserine in all meat types. The carnosine and anserine present in the human body originates from endogenous and exogenous sources. 

### 3.3. Fatty Acid Profile

The content of saturated fatty acids (SFA) C16:0 and C18:0 in dried jerky meat differed significantly depending on the species and applied additive (Table 2). The total amount of SFA was lowest in the ostrich jerky, significantly so, and highest in beef meat.

The levels of individual monounsaturated fatty acid (MUFA) (Table 3) were influenced by species (all FA), additives (C20:1) and the interaction between those factors (C16:1, C18:1c9 and C18:1c11) (Table 3). The highest levels of C16:1 were found in ostrich meat, while the lowest was found in chicken meat. Due to the particularities of the rumen activity leading to emergence of oleic acid [37], the highest levels of C18:1c9 were found in beef; nearly double as high as in poultry meat, both in the raw or dried form. Due to lower levels of oleic acid (C18:1) in monogastric animals and birds, the total amount of monounsaturated fatty acids (MUFAS) was also lower in those species. 

Fatty acids belonging to the *n*-3 group are known to have very beneficial properties for human health. Interactions between species and applied additive were found on the levels of α-linolenic acid (ALA), eicosapentaenoic acid (EPA), and docosahexaenoic acid (DHA). There was also a significant effect of the species on all *n*-3 FAs determined in the dried jerky meat (Table 4). The applied additive affected the levels of the EPA and DHA. Dried beef meat contained half the ALA as compared to chicken (Table 4). Importantly, from the human health perspective, the *n*-3 FA EPA, DPA and DHA differed significantly between species. The highest concentration of all three FAs was found in ostrich dried jerky meat (Table 4). The DHA content in ostrich meat was many times higher than in beef, and twice as high as in the chicken meat. DHA plays an important role in the brain development and functioning [38,39]. Furthermore, EPA was five-fold lower in beef, and three-fold lower in chicken dried meat as compared to ostrich. The same tendency was recorded in the case of DHA (22:6) and EPA (20:5) n3 PUFA, which were reported higher in ostrich meat as compared to beef and chicken meat [19].

Higher concentrations of the long chain *n*-3 PUFA (C:20–C:22) in dried ostrich meat resulted in the highest sum of *n*-3 PUFA in this meat (Table 4). Three-times lower *n*-3 PUFA levels were observed in beef, while half the *n*-3 PUFA were observed in chicken. Atherogenic index (AI) and thrombogenic index (TI) are based on the FA profile and indicate the risk of atherosclerosis and thrombotic diseases emergence related to consumption of food products characterised by them. In dried jerky ostrich and chicken meat, these indexes were on a very low level compared to beef meat (Table 5), as has been confirmed by previous studies on raw meat [40].

The levels of *n*-6 and *n*-3 FAs were influenced by the interaction between species and additive for the C18:2, C20:2, C20:3, C20:4 and C22:4 (Table 4 and Table 6). The highest concentration of C18:2 was recorded in dried chicken meat. This is in agreement with the research conducted by reference [6]. In turn, reference [7] compared the fatty acid profile as influenced by ostrich subspecies and they did not report significant differences among FA profile among these subspecies. 

Ostrich meat contained half of the amount found in chicken, whilst that in dry beef was nearly ten times lower. It can be speculated that in ostrich, C18:2 converts to arachidonic acid (C20:4), since the level of C20:4 was much higher than observed in chicken meat, as has been shown by previous results [41]. The *n*-6/*n*-3 ratio, also referred to as fat quality, should be around 1:1 to 4:1 [42]. In this study, ostrich meat showed significantly better proportions of *n*-6/*n*-3 as compared to chicken meat. Although beef was characterised to have an optimal *n*-6/*n*-3 ratio, it contained only trace amounts of the *n*-3 and *n*-6 FAs. In the study carried out by reference [11], dietary linseed treatment influenced and improved the *n*-6/*n*-3 ratio by lowering the value from to 6:1 to 3:1. 

The high PUFA:SFA is an important meat parameter for human health [43]. It is especially important when evaluating risks for coronary heart disease and methods of its prevention. In the current study, the more favorable proportion of PUFA:SFA was identified in ostrich and chicken meat, 0.79 and 0.83, respectively, while in beef it was only 0.09. It should be noted though that the increase in PUFA levels may cause oxidation processes in cases where there is a lack of sufficient antioxidants presence.

Therefore, it is justified to apply additives (herbs and spices) that not only help to improve the taste, but also aid to decrease oxidative processes, especially in products such as jerky with a long shelf life. The ostrich and chicken meat have shown especially high PUFA contents (Table 5), therefore to protect it from deterioration of the quality due to oxidation during storage, it is advisable to use additives.

## 4. Conclusions

Meat snacks made from ostrich, beef, and chicken meat are characterized by high concentration of nutrients including proteins, minerals (heme iron especially in ostrich, than in beef), biologically active peptides (carnosine—in beef, anserine—in ostrich then in chicken meat) and PUFA fatty acids (ostrich and chicken meat). The human health beneficial *n*-3 fatty acids levels differed significantly between species, being highest in ostrich jerky. The more favorable proportion of PUFA:SFA was identified in ostrich and chicken meat. Moreover, the atherogenic index (AI) and thrombogenic index (TI) based on the FA profile in dried jerky ostrich and chicken meat, were at a very low level, which is what is important from the consumers/human health point of view. Although these meat snacks were shown to provide plenty of biologically valuable nutrients and to have dietetic properties, further study concerning the consumers’ sensory evaluation and testing preferences for these products made from three types of meat is necessary.

## Figures and Tables

**Table 1:** Chemical composition and total hem content in dried meat (mean ± SE).

Group *	Dry Matter	Protein	Fat	Heme Iron	Carnosine	Anserine
NO	87.7 ± 0.037	76.9 ^a^ ± 1.01	4.28 ^e^ ± 0.062	948 ^a^ ± 17.7	0.367 ^e^ ± 0.001	16.8 ^a,b^ ± 0.692
SO	87.6 ± 0.007	78.1 ^a^ ± 0.067	4.32 ^e^ ± 0.159	907 ^b^ ± 1.36	0.497 ^e^ ± 0.019	18.5 ^a^ ± 0.066
SpO	84.8 ± 0.306	66.9 ^b^ ± 0.826	4.88 ^e^ ± 0.224	719 ^c^ ± 6.12	0.445 ^e^ ± 0.030	14.9 ^b^ ± 0.652
NB	81.3 ± 0.317	60.5 ^d^ ± 0.700	19.2 ^a^ ± 0.177	478 ^d^ ± 0.680	12.2 ^a^ ± 0.068	2.12 ^d^ ± 0.024
SB	81.6 ± 2.61	61.6 ^c,d^ ± 0.098	19.0 ^a^ ± 0.403	461 ^d^ ± 2.72	12.6 ^a^ ± 0.046	2.19 ^d^ ± 0.048
SpB	81.6 ± 0.051	55.7 ^e^ ± 1.45	16.5 ^b^ ± 0.241	500 ^d^ ± 8.84	10.3 ^b^ ± 0.256	1.68 ^d^ ± 0.019
NCh	80.6 ± 0.145	66.5 ^b^ ± 0.387	10.6 ^c^ ± 0.202	22.4 ^e^ ± 0.680	7.43 ^c^ ± 0.054	16.0 ^d^ ± 0.046
SCh	84.8 ± 0.057	74.2 ^a^ ± 0.071	6.92 ^d^ ± 0.138	16.3 ^e^ ± 1.36	7.07 ^c^ ± 0.304	15.5 ^b^ ± 0.364
SpCh	82.2 ± 0.158	64.5 ^b,c^ ± 0.246	7.22 ^d^ ± 0.256	49.0 ^e^ ± 4.08	5.34 ^d^ ± 0.197	12.2 ^c^ ± 0.214
**Species effect**						
Ostrich	86.7 ^a^ ± 0.603	74.0 ^a^ ± 2.28	4.49 ^c^ ± 0.143	858 ^a^ ± 44.9	0.436 ^c^ ± 0.026	16.8 ^a^ ± 0.701
Beef	81.5 ^b^ ± 0.682	59.3 ^c^ ± 1.22	18.2 ^a^ ± 0.551	480 ^b^ ± 7.49	11.7 ^a^ ± 0.458	2.00 ^c^ ± 0.103
Chicken	82.2 ^b^ ± 0.833	68.4 ^b^ ± 1.87	8.23 ^b^ ± 0.741	29.2 ^c^ ± 6.43	6.61 ^b^ ± 0.418	14.6 ^b^ ± 0.765
**Additives effect**						
Natural	83.2 ^a,b^ ± 1.44	68.0 ^b^ ± 3.06	11.3 ^a^ ± 2.73	483 ^a^ ± 169	6.67 ^a^ ± 2.17	11.6 ^a^ ± 3.02
Salt	84.7 ^a^ ± 1.29	71.3 ^a^ ± 3.14	10.1 ^b^ ± 2.86	461 ^b^ ± 163	6.73 ^a^ ± 2.22	12.1 ^a^ ± 3.18
Spices	82.5 ^b^ ± 0.737	62.4 ^c^ ± 2.19	9.55 ^c^ ± 2.25	423 ^b^ ± 125	5.36 ^b^ ± 1.80	9.60 ^b^ ± 2.56
**Source of variation**	***p*** **value**					
Species	<0.001	<0.001	<0.001	<0.001	<0.001	<0.001
Additives	0.042	<0.001	<0.001	<0.001	<0.001	<0.001
Species × Additives	0.098	0.001	<0.001	<0.001	<0.001	0.003

* NO: natural ostrich, SO: salted ostrich, SpO: spicy ostrich, NB: natural beef, SB: salted beef, SpB: spicy beef, NCh: natural chicken, SCh: salted chicken, SpCh: spicy chicken; Total hem was shown as a mg hematin/kg jerky; carnosine and anserine were shown as a mg/g jerky; ^a–e^ Means in the same column with different letters are significantly different (*p* < 0.05) separately between the group, between the species and between the additives.

**Table 2:** Saturated fatty acid composition in dried meat (%) (mean ± SE).

Group	C10:0	C12:0	C14:0	C15:0	C16:0	C17:0	C18:0	C20:0	C22:0
NO	0.191 ± 0.006	0.053 ^d^ ± 0.004	0.548 ^d^ ± 0.017	0.215 ± 0.005	19.7 ^d^ ± 0.022	0.170 ^b^ ± 0.037	9.32 ^b^ ± 0.014	0.073 ± 0.020	0.025 ± 0.009
SO	0.206 ± 0.015	0.070 ^d^ ± 0.012	0.552 ^d^ ± 0.008	0.210 ± 0.034	19.3 ^e^ ± 0.009	0.176 ^b^ ± 0.014	9.21 ^b,c^ ± 0.081	0.095 ± 0.016	0.031 ± 0.004
SpO	0.307 ± 0.031	0.086 ^d^ ± 0.007	0.553 ^d^ ± 0.011	0.231 ± 0.010	19.1 ^e^ ± 0.087	0.233 ^b^ ± 0.004	8.97 ^c^ ± 0.122	0.092 ± 0.007	0.039 ± 0.004
NB	0.074 ± 0.002	0.053 ^d^ ± 0.001	2.34 ^a^ ± 0.010	0.566 ± 0.003	25.5 ^a^ ± 0.021	0.743 ^a^ ± 0.003	11.3 ^a^ ± 0.006	0.115 ± 0.006	0.019 ± 0.002
SB	0.077 ± 0.004	0.052 ^d^ ± 0.001	2.27 ^a^ ± 0.022	0.566 ± 0.003	25.0 ^b^ ± 0.026	0.799 ^a^ ± 0.017	11.3 ^a^ ± 0.005	0.123 ± 0.001	0.031 ± 0.008
SpB	0.086 ± 0.006	0.055 ^d^ ± 0.001	2.25 ^a^ ± 0.005	0.556 ± 0.001	24.9 ^b^± 0.112	0.741 ^a^ ± 0.010	11.0 ^a^ ± 0.055	0.080 ± 0.010	0.015 ± 0.005
NCh	0.276 ± 0.013	1.65 ^b^ ± 0.002	1.66 ^c^ ± 0.036	0.179 ± 0.002	20.3 ^c^± 0.005	0.277 ^b^ ± 0.037	9.17 ^b,c^ ± 0.060	0.083 ± 0.014	0.037 ± 0.013
SCh	0.242 ± 0.011	1.86 ^a^ ± 0.009	1.79 ^b^ ± 0.028	0.151 ± 0.007	20.4 ^c^ ± 0.073	0.187 ^b^ ± 0.001	8.62 ^d^ ± 0.005	0.089 ± 0.032	0.018 ± 0.001
SpCh	0.324 ± 0.035	1.53 ^c^ ± 0.036	1.62 ^c^ ± 0.005	0.181 ± 0.007	20.3 ^c^ ± 0.002	0.182 ^b^ ± 0.021	8.94 ^c^ ± 0.021	0.081 ± 0.012	0.041 ± 0.007
**Species effect**									
Ostrich	0.234 ^b^ ± 0.025	0.069 ^b^ ± 0.007	0.551 ^c^ ± 0.006	0.219 ^b^ ± 0.010	19.4 ^c^ ± 0.103	0.193 ^b^ ± 0.016	9.17 ^b^ ± 0.076	0.086 ± 0.008	0.032 ± 0.004
Beef	0.079 ^c^ ± 0.003	0.053 ^b^ ± 0.001	2.29 ^a^ ± 0.019	0.563 ^a^ ± 0.002	25.1 ^a^ ± 0.114	0.761 ^a^ ± 0.013	11.2 ^a^ ± 0.055	0.106 ± 0.009	0.022 ± 0.004
Chicken	0.280 ^a^ ± 0.018	1.68 ^a^ ± 0.063	1.69 ^b^ ± 0.034	0.170 ^c^ ± 0.007	20.3 ^b^ ± 0.032	0.215 ^b^ ± 0.022	8.91 ^c^ ± 0.102	0.085 ± 0.010	0.032 ± 0.006
**Additives effect**									
Natural	0.180 ^b^ ± 0.037	0.584 ^b^ ± 0.336	1.52 ^a^ ± 0.331	0.320 ± 0.078	21.8 ^a^ ± 1.16	0.397 ± 0.112	9.91 ^a^ ± 0.425	0.090 ± 0.010	0.027 ± 0.005
Salt	0.175 ^b^ ± 0.032	0.662 ^a^ ± 0.380	1.54 ^a^ ± 0.324	0.309 ± 0.083	21.5 ^b^ ± 1.10	0.387 ± 0.130	9.71 ^b^ ± 0.516	0.102 ± 0.011	0.027 ± 0.004
Spices	0.239 ^a^ ± 0.050	0.556 ^b^ ± 0.307	1.47 ^b^ ± 0.313	0.322 ± 0.074	21.4 ^b^ ± 1.12	0.385 ± 0.113	9.65 ^b^ ± 0.439	0.084 ± 0.005	0.032 ± 0.006
**Source of variation**	***p*** **value**								
Species	<0.001	<0.001	<0.001	<0.001	<0.001	<0.001	<0.001	0.2314	0.1515
Additives	0.003	<0.001	0.008	0.417	<0.001	0.761	0.001	0.399	0.621
Species × Additives	0.077	<0.001	0.003	0.505	0.001	0.012	0.002	0.506	0.092

^a–e^ means in the same column with different letters are significantly different (*p* < 0.05) separately between the group, between the species and between the additives.

**Table 3:** Monounsaturated fatty acid composition in dried meat (%) (mean ± SE).

Group	C14:1	C16:1	C17:1	C18:1c9	C18:1c11	C20:1	C22:1
NO	0.110 ± 0.009	8.65 ^b^ ± 0.049	0.111 ± 0.006	24.7 ^d^ ± 0.078	3.10 ^a^ ± 0.005	0.281 ± 0.020	0.022 ± 0.006
SO	0.103 ± 0.013	8.97 ^a^ ± 0.020	0.118 ± 0.012	24.9 ^d^ ± 0.038	3.00 ^a^ ± 0.018	0.267 ± 0.033	0.025 ± 0.007
SpO	0.097 ± 0.010	8.63 ^b^ ± 0.076	0.094 ± 0.017	23.6 ^e^ ± 0.071	3.01 ^a^ ± 0.019	0.200 ± 0.006	0.032 ± 0.010
NB	0.832 ± 0.001	4.04 ^c^ ± 0.017	0.753 ± 0.006	43.8 ^a^ ± 0.029	2.64 ^b^ ± 0.040	0.109 ± 0.016	0.012 ± 0.001
SB	0.814 ± 0.001	3.95 ^c^ ± 0.006	0.811 ± 0.038	43.7 ^a^ ± 0.044	2.70 ^b^ ± 0.023	0.137 ± 0.001	0.010 ± 0.004
SpB	0.821 ± 0.001	4.03 ^c^ ± 0.013	0.793 ± 0.019	43.8 ^a^ ± 0.143	2.74 ^b^ ± 0.014	0.129 ± 0.001	0.013 ± 0.002
NCh	0.192 ± 0.034	2.58 ^d^ ± 0.035	0.136 ± 0.026	29.3 ^c^ ± 0.212	2.62 ^b^ ± 0.003	0.330 ± 0.004	0.035 ± 0.003
SCh	0.166 ± 0.006	2.74 ^d^ ± 0.019	0.131 ± 0.001	30.5 ^b^ ± 0.054	2.38 ^c^ ± 0.023	0.364 ± 0.016	0.020 ± 0.003
SpCh	0.149 ± 0.006	2.79 ^d^ ± 0.039	0.091 ± 0.002	30.0 ^b^ ± 0.062	2.60 ^b^ ± 0.075	0.305 ± 0.018	0.029 ± 0.008
**Species effect**							
Ostrich	0.1 ^c^ ± 0.005	8.75 ^a^ ± 0.073	0.11 ^b^ ± 0.007	24.4 ^c^ ± 0.250	3.04 ^a^ ± 0.023	0.25 ^b^ ± 0.019	0.03 ^a^ ± 0.004
Beef	0.8 ^a^ ± 0.003	4.01 ^b^ ± 0.019	0.79 ^a^ ± 0.015	43.8 ^a^ ± 0.041	2.69 ^b^ ± 0.023	0.13 ^c^ ± 0.007	0.01 ^b^ ± 0.001
Chicken	0.17 ^b^ ± 0.012	2.7 ^c^ ± 0.041	0.12 ^b^ ± 0.011	29.9 ^b^ ± 0.236	2.53 ^c^ ± 0.053	0.33 ^a^ ± 0.013	0.03 ^a^ ± 0.004
**Additives effect**							
Natural	0.378 ± 0.145	5.09 ^b^ ± 1.16	0.333 ± 0.133	32.6 ^b^ ± 3.64	2.79 ^a^ ± 0.101	0.240 ^a,b^ ± 0.043	0.023 ± 0.005
Salt	0.361 ± 0.144	5.22 ^a^ ± 1.21	0.353 ± 0.145	33.0 ^a^ ± 3.53	2.69 ^b^ ± 0.113	0.256 ^a^ ± 0.043	0.019 ± 0.003
Spices	0.356 ± 0.147	5.15 ^a,b^ ± 1.12	0.326 ± 0.148	32.5 ^b^ ± 3.76	2.78 ^a^ ± 0.078	0.211 ^b^ ± 0.033	0.025 ± 0.005
**Source of variation**	***p*** **value**						
Species	<0.001	<0.001	<0.001	<0.001	<0.001	<0.001	0.011
Additives	0.168	0.007	0.212	<0.001	0.008	0.023	0.424
Species × Additives	0.734	0.001	0.266	<0.001	0.006	0.099	0.528

^a–e^ means that values in the same column with different letters are significantly different (*p* < 0.05) separately between the group, between the species and between the additives.

**Table 4:** Polyunsaturated fatty acid *n*-3 group composition in dried meat (%) (mean ± SE).

Group	C18:3n3	C20:3n3	C20:4n3	C20:5n3 (EPA)	C22:5n3	C22:6n3 (DHA)
NO	1.07 ^c,d^ ± 0.021	0.061 ± 0.016	0.065 ± 0.004	0.516 ^a,b^ ± 0.000	0.893 ± 0.027	0.649 ^a^ ± 0.039
SO	1.00 ^d^ ± 0.007	0.055 ± 0.013	0.026 ± 0.009	0.537 ^a^ ± 0.001	0.812 ± 0.035	0.588 ^a,b^ ± 0.015
SpO	1.06 ^c,d^ ± 0.009	0.049 ± 0.002	0.052 ± 0.011	0.423 ^b^ ± 0.007	0.854 ± 0.011	0.626 ^a^ ± 0.036
NB	0.477 ^e^ ± 0.006	0.031 ± 0.007	0.065 ± 0.007	0.093 ^d^ ± 0.012	0.209 ± 0.010	0.031 ^e^ ± 0.010
SB	0.477 ^e^ ± 0.016	0.020 ± 0.011	0.063 ± 0.003	0.104 ^c,d^ ± 0.006	0.231 ± 0.015	0.033 ^e^ ± 0.005
SpB	0.547 ^e^ ± 0.017	0.017 ± 0.003	0.053 ± 0.019	0.085 ^d^ ± 0.007	0.226 ± 0.019	0.037 ^e^ ± 0.003
NCh	1.19 ^b,c^ ± 0.001	0.074 ± 0.017	0.083 ± 0.020	0.190 ^c^ ± 0.002	0.621 ± 0.015	0.362 ^c,d^ ± 0.021
SCh	1.35 ^a^ ± 0.074	0.073 ± 0.001	0.053 ± 0.005	0.137 ^c,d^ ± 0.017	0.557 ± 0.019	0.312 ^d^ ± 0.002
SpCh	1.23 ^a,b^ ± 0.020	0.055 ± 0.016	0.097 ± 0.007	0.145 ^c,d^ ± 0.045	0.616 ± 0.020	0.465 ^b,c^ ± 0.033
**Species effect**						
Ostrich	1.05 ^b^ ± 0.015	0.06 ^a^ ± 0.006	0.05 ^b^ ± 0.008	0.49 ^a^ ± 0.022	0.86^a^ ± 0.019	0.62 ^a^ ± 0.018
Beef	0.5 ^c^ ± 0.016	0.02 ^b^ ± 0.004	0.06 ^a,b^ ± 0.006	0.09 ^c^ ± 0.005	0.22^c^ ± 0.008	0.03 ^c^ ± 0.003
Chicken	1.26 ^a^ ± 0.036	0.07 ^a^ ± 0.007	0.08 ^a^ ± 0.010	0.16 ^b^ ± 0.016	0.59^b^ ± 0.015	0.38 ^b^ ± 0.030
**Additives effect**						
Natural	0.912 ± 0.140	0.055 ± 0.010	0.071 ± 0.007	0.266 ^a^ ± 0.081	0.574 ± 0.126	0.347 ^a,b^ ± 0.114
Salt	0.943 ± 0.161	0.049 ± 0.011	0.047 ± 0.007	0.259 ^a^ ± 0.088	0.534 ± 0.107	0.311 ^b^ ± 0.101
Spices	0.948 ± 0.131	0.040 ± 0.009	0.067 ± 0.011	0.218 ^a^ ± 0.067	0.565 ± 0.116	0.376 ^a^ ± 0.112
**Source of variation**	***p*** **value**					
Species	<0.001	0.003	0.029	<0.001	<0.001	<0.001
Additives	0.294	0.313	0.057	0.014	0.082	0.021
Species x Additives	0.017	0.969	0.224	0.032	0.178	0.047

^a–e^ means that values in the same column with different letters are significantly different (*p* < 0.05) separately between the group, between the species and between the additives.

**Table 5:** Sum of fatty acid, atherogenic index (AI) and thrombogenic index (TI) in dried meat (%) (mean ± SE).

Group	SFA	MUFA	PUFAn6	PUFAn3	PUFA n6/n3	AI	TI
NO	30.3 ± 0.054	37 ^b,c^ ± 0.13	20 ^d^ ± 0.1	3.25 ± 0.08	6.15 ^c^ ± 0.11	0.36 ^e^ ± 0.002	0.67 ^d^ ± 0.002
SO	29.8 ± 0.06	37.4 ^b^ ± 0.08	19.7 ^d^ ± 0.15	3.02 ± 0.05	6.52 ^b,c^ ± 0.06	0.36 ^e^ ± 0.001	0.66 ^d,e^ ± 0.004
SpO	29.6 ± 0.045	35.7 ^d,e^ ± 0.03	21.8 ^c^ ± 0.051	3.07 ± 0.013	7.09 ^b^ ± 0.013	0.354 ^e^ ± 0.003	0.636 ^f^ ± 0.000
NB	40.6 ± 0.035	52.2 ^a^ ± 0.029	2.49 ^e^ ± 0.025	0.905 ± 0.025	2.75 ^d^ ± 0.102	0.628 ^a^ ± 0.001	1.19 ^a^ ± 0.000
SB	40.2 ± 0.017	52.2 ^a^ ± 0.015	2.51 ^e^ ± 0.007	0.928 ± 0.012	2.70 ^d^ ± 0.028	0.613 ^b^ ± 0.002	1.17 ^b^ ± 0.002
SpB	39.7 ± 0.162	52.3 ^a^ ± 0.151	2.85 ^e^ ± 0.006	0.965 ± 0.031	2.96 ^d^ ± 0.087	0.606 ^b^ ± 0.001	1.14 ^c^ ± 0.003
NCh	33.6 ± 0.048	35.2 ^e^ ± 0.251	25.5 ^a^ ± 0.064	2.52 ± 0.037	10.1 ^a^ ± 0.172	0.452 ^c,d^ ± 0.004	0.648 ^e,f^ ± 0.002
SCh	33.3 ± 0.086	36.3 ^c,d^ ± 0.04	25.0 ^b^ ± 0.029	2.48 ± 0.036	10.1 ^a^ ± 0.158	0.461 ^c^ ± 0.000	0.640 ^f^ ± 0.000
SpCh	33.2 ± 0.128	35.9 ^d^ ± 0.155	25.0 ^b^ ± 0.112	2.61 ± 0.069	9.58 ^a^ ± 0.211	0.445 ^d^ ± 0.003	0.644 ^f^ ± 0.002
**Species effect**							
Ostrich	29.9 ^c^ ± 0.118	36.7 ^b^ ± 0.324	20.5 ^b^ ± 0.409	3.11 ^a^ ± 0.051	6.59 ^b^ ± 0.176	0.359 ^c^ ± 0.002	0.653 ^b^ ± 0.006
Beef	40.2 ^a^ ± 0.169	52.2 ^a^ ± 0.045	2.61 ^c^ ± 0.075	0.933 ^c^ ± 0.02	2.80 ^c^ ± 0.061	0.615 ^a^ ± 0.004	1.17 ^a^ ± 0.009
Chicken	33.4 ^b^ ± 0.085	35.8 ^c^ ± 0.228	25.1 ^a^ ± 0.108	2.54 ^b^ ± 0.033	9.92 ^a^ ± 0.134	0.453 ^b^ ± 0.003	0.644 ^c^ ± 0.002
**Additives effect**							
Natural	34.8 ^a^ ± 1.93	41.4 ^b^ ± 3.42	16.0 ^b^ ± 4.38	2.23 ± 0.439	6.33 ± 1.34	0.481 ^a^ ± 0.049	0.835 ^a^ ± 0.113
Salt	34.5 ^b^ ± 1.92	41.9 ^a^ ± 3.24	15.7 ^c^ ± 4.29	2.14 ± 0.397	6.43 ± 1.35	0.478 ^a^ ± 0.047	0.824 ^b^ ± 0.111
Spices	34.2 ^c^ ± 1.87	41.3 ^b^ ± 3.47	16.5 ^a^ ± 4.37	2.21 ± 0.405	6.54 ± 1.22	0.468 ^b^ ± 0.047	0.808 ^c^ ± 0.106
**Source of variation**	***p*** **value**						
Species	<0.001	<0.001	<0.001	<0.001	<0.001	<0.001	<0.001
Additives	<0.001	<0.001	<0.001	0.092	0.166	<0.001	<0.001
Species x Additives	0.140	<0.001	<0.001	0.050	0.002	0.006	<0.001

AI: atherogenic index ((C12:0 + 4 × C14:0 + C16:0)/(∑MUFA + ∑PUFA)); TI: thrombogenic index ((C14:0 + C16:0 + C18:0)/(0.5 × ∑MUFA + 0.5 × ∑PUFA *n*-6 + 3 × PUFA *n*-3 + PUFA *n*-3/*n*-6) (Ulbricht & Southgate. 1991); ^a–f^ means that values in the same column with different letters are significantly different (*p* < 0.05) separately between the group, between the species and between the additives.

**Table 6:** Polyunsaturated fatty acid *n*-6 group composition in dried meat (%) (mean ± SE).

Group	C18:2n6	C18:3n6	C20:2n6	C20:3n6	C20:4n6	C22:4n6	C22:5n6
NO	10.2 ^e^ ± 0.026	0.024 ± 0.010	0.42 ^a^ ± 0.065	0.384 ^c^ ± 0.033	7.83 ^b^ ± 0.027	0.881 ^b^ ± 0.009	0.216 ± 0.013
SO	10.2 ^e^ ± 0.045	0.028 ± 0.009	0.5 ^a^ ± 0.031	0.399 ^c^ ± 0.022	7.55 ^b^ ± 0.058	0.876 ^b^ ± 0.047	0.196 ± 0.022
SpO	11.1 ^d^ ± 0.151	0.017 ± 0.004	0.4 ^a^ ± 0.019	0.314 ^c,d^ ± 0.013	8.70 ^a^ ± 0.158	1.05 ^a^ ± 0.023	0.224 ± 0.010
NB	1.66 ^f^ ± 0.016	0.134 ± 0.014	0.03 ^b^ ± 0.010	0.178 ^e^ ± 0.003	0.42 ^e^ ± 0.001	0.043 ^b^ ± 0.005	0.018 ± 0.002
SB	1.66 ^f^ ± 0.001	0.140 ± 0.012	0.04 ^b^ ± 0.003	0.18 ^e^ ± 0.018	0.41 ^e^ ± 0.008	0.054 ^b^ ± 0.003	0.019 ± 0.007
SpB	1.92 ^f^ ± 0.002	0.128 ± 0.011	0.03 ^b^ ± 0.013	0.188 ^d,e^ ± 0.012	0.48 ^e^ ± 0.020	0.053 ^b^ ± 0.014	0.044 ± 0.000
NCh	19.9 ^b^ ± 0.072	0.025 ± 0.001	0.54 ^a^ ± 0.026	0.718 ^a^ ± 0.028	3.27 ^c^ ± 0.040	0.865 ^b,c^ ± 0.018	0.166 ± 0.032
SCh	20.4 ^a^ ± 0.030	0.027 ± 0.009	0.4 ^a^ ± 0.002	0.541 ^b^ ± 0.013	2.73 ^d^ ± 0.031	0.727 ^c^ ± 0.005	0.125 ± 0.040
SpCh	19.3 ^c^ ± 0.021	0.038 ± 0.010	0.43 ^a^ ± 0.010	0.619 ^a,b^ ± 0.039	3.46 ^c^ ± 0.026	0.946 ^a,b^ ± 0.047	0.179 ± 0.036
**Species effect**							
Ostrich	10.5 ^b^ ± 0.187	0.02 ^b^ ± 0.004	0.44 ^a^ ± 0.028	0.377 ^b^ ± 0.020	8.03 ^a^ ± 0.222	0.94 ^a^ ± 0.039	0.21 ^a^ ± 0.009
Beef	1.75 ^c^ ± 0.056	0.13 ^a^ ± 0.006	0.03 ^b^ ± 0.004	0.18 ^c^ ± 0.006	0.44 ^c^ ± 0.015	0.05 ^c^ ± 0.004	0.03 ^c^ ± 0.006
Chicken	19.9 ^a^ ± 0.204	0.03 ^b^ ± 0.004	0.45 ^a^ ± 0.027	0.63 ^a^ ± 0.035	3.15 ^b^ ± 0.138	0.85 ^b^ ± 0.043	0.16 ^b^ ± 0.019
**Additives effect**							
Natural	10.6 ^b^ ± 3.33	0.061 ± 0.024	0.327 ± 0.097	0.427 ^a^ ± 0.100	3.84 ^b^ ± 1.37	0.596 ^b^ ± 0.175	0.133 ± 0.039
Salt	10.7 ^a^ ± 3.43	0.065 ± 0.024	0.311 ± 0.090	0.374 ^b^ ± 0.066	3.57 ^c^ ± 1.33	0.552 ^b^ ± 0.160	0.113 ± 0.035
Spices	10.8 ^a^ ± 3.18	0.061 ± 0.022	0.285 ± 0.081	0.373 ^b^ ± 0.082	4.21 ^a^ ± 1.52	0.684 ^a^ ± 0.201	0.149 ± 0.036
**Source of variation**	***p*-value**						
Species	<0.001	<0.001	<0.001	<0.001	<0.001	<0.001	<0.001
Additives	0.012	0.848	0.216	0.028	<0.001	<0.001	0.214
Species x Additives	<0.001	0.700	0.024	0.008	<0.001	0.007	0.905

^a–f^ means that values in the same column with different letters are significantly different (*p* < 0.05) separately between the group, between the species and between the additive.
